# Draft genome sequence of endophyte *Bacillus amyloliquefaciens* strain Apn01R, isolated from the roots of *Alhagi pseudalhagi* (Bieb.) Fisch

**DOI:** 10.1128/mra.01074-24

**Published:** 2024-12-17

**Authors:** Vladimir K. Chebotar, Anna V. Myskova, Margarita Khudyaeva, Oksana V. Keleinikova, Maria E. Baganova, Alexander N. Zaplatkin, Veronika N. Pishchik, Elena P. Chizhevskaya

**Affiliations:** 1 All-Russia Research Institute for Agricultural Microbiology, Pushkin, Russia; 2 Department of Genetics and Biotechnology, Faculty of Biology, Saint Petersburg State University, Saint Petersburg, Russia; California State University San Marcos, San Marcos, California, USA

**Keywords:** Whole-genome, sequence, endophyte, *Bacillus amyloliquefaciens*

## Abstract

In this article, we announce the sequence and draft analysis of the genome of endophyte *Bacillus amyloliquefaciens* strain Apn01R, isolated from the roots of *Alhagi pseudalhagi* (Bieb.) Fisch. Our results show genes that may be crucial to defy abiotic stress and repel bacterial and fungal pathogens.

## ANNOUNCEMENT

*Bacillus* genus of Gram-positive spore-forming bacteria (phylum Firmicutes) is renowned for species capable of synthesizing antibiotics, vitamins, and other secondary metabolites (1). These substances can be helpful not only to the bacteria but also to their hosts. In particular, plant-associated species *Bacillus amyloliquefaciens* is promising for agriculture, as it can improve nutrient availability and secrete organic growth factors (e.g., auxin) (2). Another aspect is that introducing the species can modify the soil microbial community beneficially (3).

The Alhagi plants were collected during an expedition across the Stavropol region of Russia near Neftekumsk city (44.4502°N, 44.5958°E). The Apn01R strain was isolated from the roots of *Alhagi pseudalhagi* plant host according to the protocol (4). Plant roots were disinfected with tap water for 30 s followed by 70% ethanol for 5 min, then with 15% H_2_O_2_ for 10 min and sterile water for 2 min five times, and were subsequently crushed with a pestle in a mortar under sterile conditions. Aliquots of 100 mL of the resulting plant juices were plated on 1/20 tryptic soy agar (TSA, Difco Laboratories, Michigan, USA) plates. Strain Apn01R was grown in Luria-Bertani (LB) medium (Thermo Fisher Scientific, Massachusetts, USA) at 30°C overnight. Bacterial DNA was extracted with Wizard Genomic DNA Purification Kit (Promega, USA) and was prepared for sequencing using the KAPA HyperPlus kit (Roche, Switzerland). Whole-genome sequencing was performed using the NextSeq 1000 platform (Illumina, USA) with the NextSeq 1000/2000 P1 Reagents (300 cycles), resulting in paired reads. Sequencing was performed at the Lopukhin center “Genomics, Proteomics, Metabolomics” in Russia.

For any further software applications, default parameters were used except where otherwise noted. A total of 2,245,904 reads were generated with an average length of 150 bp. The sequence quality was estimated using FastQC v. 0.11.9 (5). *De novo* genome assembly was performed with SPAdes v.3.13.1 using the “careful” option (6). The draft genome assembly amounts to 3,870,665 bp and contains 10 scaffolds (average coverage: 226.437×). We assessed the assembly quality with QUAST v. 5.2.0 (7), resulting in N50 = 3,855,457 and GC% = 46.40. The BUSCO v5.2.2 (8) results recovered 99.8% of the Bacillales database. In total, 3,720 coding genes and 103 RNA genes were found by running Prokka v. 1.14.5 (9). PGAP (10) was used as an additional annotation tool. Then, with the help of FastANI software v1.34 (11), we determined that the isolated strain belongs to the *B. amyloliquefaciens* species (average nucleotide identity of 98.78% with reference genome ASM1939692v1).

We searched for biosynthetic gene clusters using antiSMASH v. 7.1.0 (12). Additionally, we were able to identify several groups of stress-response genes, e.g., related to salt and hydroperoxide stress, and a variety of antimicrobial agents. The strain’s ability to synthesize polyketides allows us to speculate about its wide range of adaptive secondary metabolites aside from those highlighted in our study. In Table 1, we list genes and gene clusters potentially involved in adaptive responses.

**TABLE 1:** Selection of annotated genes’ products that can be involved in adaptive responses

Anti-fungal activity	Plipastatin/fengycin cluster
Antibiotic synthesis and resistance	Bacitracin transport ATP-binding protein BcrA
	Bicyclomycin resistance protein
	Linear gramicidin dehydrogenase LgrE
	Linear gramicidin synthase
	Penicillin-binding protein H
	Plantazolicin cluster
Stress resistance	Stress-response proteins YkoL, YvgO, CsbD, SCP2, YhaX, NhaX
	Stress-response kinase A
	Salt-stress responsive protein YocM
	Organic peroxide resistance transcriptional regulator
	Organic peroxide resistance protein OhrB
	Arsenical pump membrane protein
Multidrug resistance	Multidrug export protein EmrB
	Multidrug resistance protein MdtB
Other	Polyketide synthase
	Surfactin synthase

## Data Availability

The draft genome sequences and raw reads were deposited in National Center for Biotechnology Information (NCBI) and can be accessed under BioProject PRJNA1155019, SRA SRR30503713, and assembly GCA_041947065.1. Genome annotations obtained using Prokka and antiSMASH software are available on Figshare: https://doi.org/10.6084/m9.figshare.27301482.v1.
